# CD14^-^ CD16^+^ monocyte PD-L1 prevents early tuberculosis progression and constrains reactivation under immune checkpoint therapy

**DOI:** 10.3389/fcimb.2025.1684030

**Published:** 2025-12-09

**Authors:** Wentao Wang, Chenyang Liu, Wenxaio Wu, Zhengjun Yi, Jie Shen

**Affiliations:** 1 School of Laboratory Medicine, Shandong Second Medical University, Weifang, China; 2 Shandong Advanced Academy Engineering Research Institute for Precision Medicine Innovation and Transformation of Infectious Disease, Weifang, China; 3 Affiliated Hospital of Shandong Second Medical University, Weifang, China

**Keywords:** latent tuberculosis infection, PD-L1, CD14- CD16+ monocyte, immune checkpoint, transcriptional regulation

## Abstract

Immune checkpoint blockade (ICB) has revolutionized cancer therapy, yet its unintended impact on chronic infections remains poorly understood. Here, we identify PD-L1 expression on CD14^-^ CD16^+^ monocytes as a critical determinant for preventing early tuberculosis (TB) progression. By integrating real-world adverse event data, Mendelian randomization (MR), and transcriptomic analysis, we establish a causal and cell-specific link between PD-L1 downregulation and early TB progression. Analysis of the FAERS database revealed that the PD-L1 inhibitor durvalumab is significantly associated with TB-related adverse events (reporting odds ratio = 7.81; 95% CI: 4.43-13.78; *P* = 1.10×10^-18^). MR analysis confirmed that genetically elevated PD-L1 expression in CD14^-^ CD16^+^ monocytes confers protection against early TB progression (OR = 0.918, *P* = 0.042), independent of confounding or reverse causality. Transcriptomic profiling revealed that PD-L1^high^ CD14^-^ CD16^+^ monocytes exhibit enhanced antigen surveillance, whereas PD-L1^low^ cells display metabolic reprogramming associated with immune escape. Upstream regulatory analysis identified CDAN1, TCOF1, and LMO2 as transcriptional drivers of PD-L1, enriched in high-risk individuals. In *silico* drug prediction and molecular docking suggested several PD-L1-modulating compounds, including ruthenium, pomalidomide, zidovudine, and lycorine. Notably, several of these compounds (e.g., ruthenium complexes, pomalidomide, aziridine) have reported anti-mycobacterial effects, which supports the reliability of our screening results and their potential relevance to TB regulation. *In vitro* validation demonstrated that lycorine dose-dependently upregulates PD-L1 and inhibits *Mycobacterium tuberculosis* reactivation. Together, our findings define a mechanistic axis in CD14^-^ CD16^+^ monocytes that underpins early TB control and is vulnerable to PD-L1 blockade. Collectively, these findings align with the established notion that assessing latent tuberculosis infection before initiating immune-modulating therapies is essential for minimizing reactivation risk, and propose tractable molecular targets for preventing TB reactivation in immunocompromised hosts.

## Graphical Abstract

**Figure:**
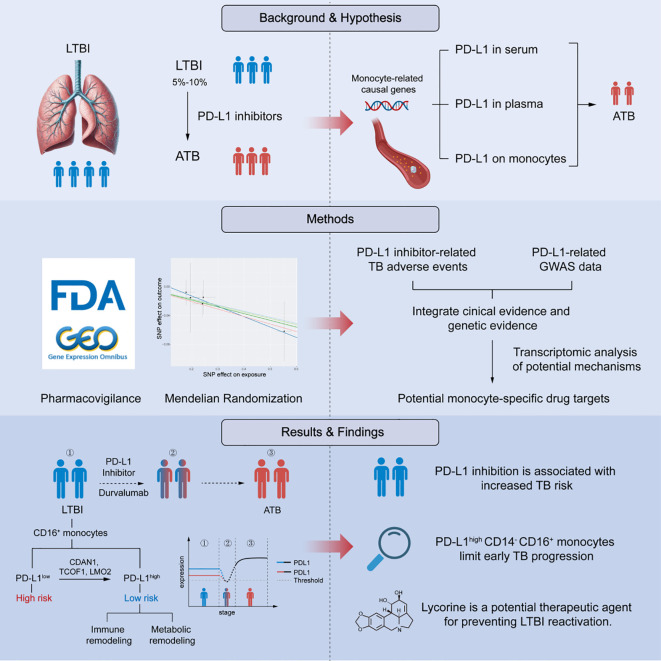

## Introduction

Latent tuberculosis infection (LTBI) affects nearly one-quarter of the global population and serves as a major reservoir for future active tuberculosis (TB) cases, particularly under conditions of immune suppression (Getahun et al., 2015; Fehily et al., 2022). The immune system plays a pivotal role in maintaining the dormancy of *Mycobacterium tuberculosis* (Mtb), yet the cellular and molecular mechanisms that sustain latency and how these mechanisms fail during reactivation remain poorly defined (Boom et al., 2021).

The advent of immune checkpoint inhibitors (ICIs) has revolutionized cancer therapy but has also unveiled the unintended risk of latent infection reactivation. In particular, antibodies targeting programmed death-ligand 1 (PD-L1) reinvigorate anti-tumor T-cell responses but may inadvertently disrupt immune homeostasis against chronic pathogens (Kanjanapan and Yip, 2020). Reports of TB reactivation in patients treated with anti-PD-L1 therapy have raised significant clinical concern (Anastasopoulou et al., 2019; Liu et al., 2022).

However, whether this risk arises from the immunomodulatory effects of PD-L1 blockade itself or from confounding factors such as diabetes or prior immunosuppressive therapy remains uncertain (Ahmed et al., 2022; Franco et al., 2024). Notably, unlike conventional immunosuppressants, ICIs do not directly suppress immune function but instead reshape immune checkpoint signaling. Several pathological observations have documented TB reactivation following PD-L1 blockade even in the absence of corticosteroid or other immunosuppressive exposure, suggesting a distinct immunopathological basis for this phenomenon (Liu et al., 2022). Despite accumulating pharmacovigilance signals, definitive mechanistic evidence establishing a causal relationship between PD-L1 inhibition and TB reactivation is still lacking.

Human monocytes are divided into three major subsets—classical (CD14^+^ CD16^-^), intermediate (CD14^+^ CD16^+^), and nonclassical (CD14^-^ CD16^+^)—each contributing differently to immune defense during TB infection (Sampath et al., 2018). Nonclassical monocytes, which constitute 2-11% of circulating monocytes, exhibit proinflammatory characteristics, secrete cytokines in response to infection, and participate in antigen presentation and T-cell activation. Recent evidence suggests that this subset may drive the progression from latent to active TB (Ma et al., 2025); however, their precise functional role during early infection remains elusive. Given that PD-L1 expression on monocytes dynamically changes during Mtb infection and influences bacterial containment and host outcome (Pan et al., 2022), we hypothesized that PD-L1 on CD14^-^ CD16^+^ monocytes acts as a regulatory barrier that maintains latency and prevents reactivation.

To test this hypothesis, we integrated real-world pharmacovigilance data, Mendelian randomization (MR) analysis, and transcriptomic profiling to investigate the cell-specific role of PD-L1 in early TB progression. We found that high PD-L1 expression on CD14^-^ CD16^+^ monocytes is associated with protection against TB reactivation, independent of systemic PD-L1 levels. Conversely, reduced PD-L1 expression corresponds to transcriptional signatures of immune evasion and metabolic reprogramming, indicative of a permissive state for reactivation. Further, we identified TCOF1, LMO2, and CDAN1 as upstream regulators of PD-L1 expression, enriched in individuals at higher risk of progression. Through in silico drug screening and molecular docking, we proposed candidate compounds capable of modulating this pathway and validated their effects *in vitro*.

Together, our findings establish a causal and cell-specific link between PD-L1 expression and early TB progression, uncovering an immunoregulatory axis within CD14^-^ CD16^+^ monocytes that safeguards against reactivation. This study provides mechanistic insight into the host-pathogen equilibrium that defines latency and offers a conceptual framework for precision assessment of infection risk in the context of immune checkpoint therapy.

## Methods

### Data sources

Bulk RNA-sequencing data were obtained from the GEO (Gene Expression Omnibus) database (https://www.ncbi.nlm.nih.gov/geo/). Specifically, the GSE185372 dataset, which includes RNA-seq profiles from 294 monocyte samples (Hillman et al., 2022), was used in this study. Active TB (ATB) was defined by clinical and/or radiological evidence of pulmonary tuberculosis with microbiological confirmation by M. tuberculosis-specific molecular testing on sputum. Latent TB infection (LTBI) was defined by a positive interferon-γ release assay (IGRA) in the absence of clinical or radiological signs of disease. All participants were HIV negative.

For Mendelian randomization (MR) analysis, genetic instruments associated with PD-L1 expression on monocytes were obtained from genome-wide association studies (GWAS) in the IEU Open GWAS database. The cohort comprised 3, 757 individuals with flow cytometry-based phenotyping of monocyte subsets and surface markers (Orrù et al., 2020). GWAS summary statistics for circulating PD-L1 levels in plasma (n=3, 301) and serum (n=5, 366) were obtained from previously published datasets by Sun et al. and Gudjonsson et al., respectively (Sun et al., 2018; Gudjonsson et al., 2022).

Genetic variants associated with tuberculosis early progression were extracted from a large-scale GWAS conducted in Lima, Peru (n = 3, 980) (Luo et al., 2019). In this cohort, early progressors were defined as individuals who developed microbiologically confirmed TB disease within one year after exposure to an index TB case, whereas non-progressors were household contacts with a positive tuberculin skin test (TST) but without clinical disease during 12 months of follow-up.

No additional ethical approval or informed consent was required, as this study used publicly available datasets from established databases. For additional information on data sources, refer to **Supplementary Table 1**.

### FAERS data processing and signal mining

**Figure 1:**
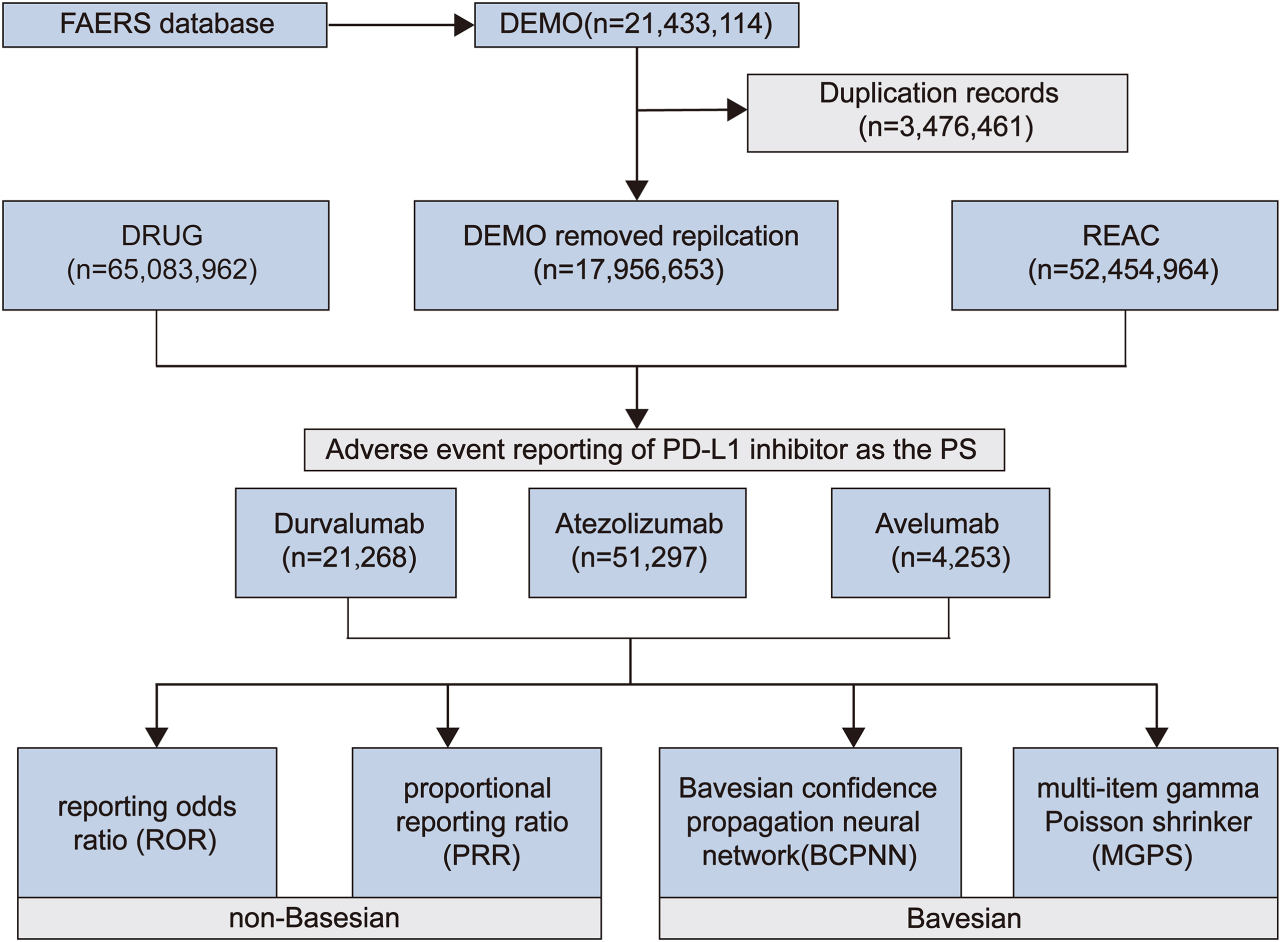
FAERS Data analysis workflow.

We conducted pharmacovigilance research on TB-related adverse events (AEs) associated with PD-L1 inhibitors using the FAERS database (https://www.fda.gov/drugs/drug-approvals-and-databases/fda-adverse-event-reporting-system-faers-database). Data were obtained from the FAERS public dashboard, covering reports on PD-L1 inhibitors (atezolizumab, avelumab, and durvalumab) from Q1–2004 to Q2 2024. To enhance data reliability, we followed the FDA’s recommended procedures, removing any potential duplicate reports. Using the Medical Dictionary for Regulatory Activities (MedDRA, version 26.0), we identified all cases of TB-related adverse reactions linked to anti-PD-L1 inhibitors. These cases were defined by adverse event descriptions such as “Pulmonary Tuberculosis” or “Tuberculosis”. The extracted data included patient demographics (age, gender), report type, medication details (monotherapy or combination therapy), type of adverse reaction, and outcome. To further assess the strength of these adverse event signals, we calculated several pharmacovigilance metrics, including the Reporting Odds Ratio (ROR) (Caster et al., 2020; Zhou et al., 2023), Proportional Reporting Ratio (PRR) (Krantz et al., 2023), Empirical Bayesian Geometric Mean (EBGM) (Xi et al., 2024), and Information Component (IC) (Zhou et al., 2024), which are widely used for signal detection in pharmacovigilance. The formulas and interpretations for these signal detection methods can be found in **Table 1**. Additionally, chi-square tests were applied to the positive signals to assess statistical significance, with the Benjamini-Hochberg (BH) correction used to control for false positives in multiple comparisons. A corrected *p*-value (adjusted *p*)< 0.05 was considered statistically significant. The extraction and analysis process strictly adhered to established methods and standard operating procedures in the pharmacovigilance field. The detailed workflow is shown in **Figure 1**.

**Table 1:** Four major algorithms used for signal detection.

Algorithms	Equation	Criteria
ROR (Sun et al., 2018)	ROR= (a/c)/(b/d)	95% CI>1, N≥3
	$95\%\;CI=\;e^{\ln(ROR)\pm 1.96(\frac{1}{a}+\;\frac{1}{b}+\;\frac{1}{c}+\;\frac{1}{d})\;\frac{1}{2}}$	
PRR (Krantz et al., 2023)	PRR= [a/(a+b)]/[c/(c+d)]	PRR≥2, *χ^2^*≥4, N≥3
	*χ^2^*= [(ad-bc)^2](a+b+c+d)/[(a+b)(c+d)(a+c)(b+d)]	
BCPNN (Xi et al., 2024)	IC= log_2_a(a+b+c+d)/[(a+c)(a+b)]	IC_025_>0
	$IC_{025}=\;e^{\ln(IC)-1.96(\frac{1}{a}+\;\frac{1}{b}+\;\frac{1}{c}+\;\frac{1}{d})\;\frac{1}{2}}$	
MGPS (Zhou et al., 2024)	EBGM=a(a+b+c+d)/[(a+c)(a+b)]	EBGM_05_>2
	$EBGM_{05}=\;e^{\ln(EBGM)-1.64(\frac{1}{a}+\;\frac{1}{b}+\;\frac{1}{c}+\;\frac{1}{d})\;\frac{1}{2}}$	

a, number of reports containing both the target drug and target adverse reaction reports; b, number of reports containing other adverse reaction reports of the target drug; c, number of reports containing the target adverse reaction reports of other drugs; d, number of reports containing other drugs and other adverse reaction reports. 95%CI, 95% confidence interval; N, the number of reports; *χ^2^*, chi-squared; EBGM, empirical Bayesian geometric mean; EBGM_05_, the lower limit of 95% CI of EBGM; IC, information component; IC_025_, the lower limit of 95% CI of the IC. ROR, reporting odds ratio; PRR, proportional reporting ratio; MGPS, multi-item gamma poisson shrinker; BCPNN, Bayesian confidence propagation neural network.

### MR analysis

For MR analyses, immunophenotype instrumental variables (IVs) were selected based on the following criteria: a threshold of 1×10–^2^ to ensure a sufficient number of IVs, linkage disequilibrium (*R^2^*)< 0.001, clumping distance of 10, 000 kb, and F-statistics > 10, which are commonly recommended in MR analyses (Wang et al., 2024; Wu et al., 2024; Yang et al., 2024). The selection criteria for IVs in reverse MR are the same. To ensure that the IVs were not associated with confounding factors (**Supplementary Table 2**) related to TB, we cross-referenced them using the IEU database (https://gwas.mrcieu.ac.uk/) and excluded any relevant SNPs.

In this MR study, the Inverse Variance Weighting (IVW) method was used to estimate the overall effect size. Given the limited number of available instrumental variables, the IVW approach was adopted as the primary estimator, as it provides unbiased and efficient causal estimates under the assumption that all instruments are valid. To evaluate the robustness of causal inference, complementary methods (MR-Egger regression, weighted median, simple mode, and weighted mode) were applied to assess the consistency of causal direction, following the recommendation by Bowden et al (Burgess et al., 2017; Porcu et al., 2019). The robustness of IVW results was further examined through heterogeneity and pleiotropy tests using MR-PRESSO. A minimum of three SNPs is required for complementary methods, and at least two SNPs for IVW. For traits with only one instrumental variable, the Wald ratio method was used for evaluation (Boehm and Zhou, 2022). MR-PRESSO was also used as a major supplementary tool for positive MR results (Verbanck et al., 2018). Sensitivity analyses included: Cochran’s Q statistic to assess heterogeneity (Hemani et al., 2018); MR-Egger regression to estimate horizontal pleiotropy via its intercept (Bowden et al., 2015); MR-PRESSO for detecting horizontal pleiotropy (Verbanck et al., 2018); A leave-one-out analysis to identify any single SNP that disproportionately influenced the results; 5.MR-PRESSO outlier test to detect outliers (Verbanck et al., 2018).

As this is an exploratory study, no correction for multiple testing was applied. All statistical analyses were two-sided, with a significance level of 0.05, and were conducted using the ‘TwoSampleMR’ R package and R version 4.3.2 for all computations.

### Bulk transcriptomic analysis

Bulk RNA sequencing data were normalized using the DESeq2 package (v1.44.0) in RStudio (v4.4.1), with genes exhibiting more than 50% zero expression across samples being excluded from the analysis (Love et al., 2014). Differential expression analysis was performed using DESeq2, and differentially expressed genes (DEGs) were sorted based on log2 fold change (logFC), generating a gene list for downstream Gene Set Enrichment Analysis (GSEA). For the single-gene GSEA, the LTBI gene expression matrix was used. Samples with target gene expression higher or lower than the median were assigned to the high or low expression groups, respectively. The logFC between the high and low expression groups was computed, and genes were ranked in descending order based on their logFC values. GSEA for Gene Ontology (GO) analysis was conducted using the clusterProfiler package (v4.12.6), with gene sets filtered by a *p*-value< 0.05 and an absolute normalized enrichment score (|NES|) > 1. Gene sets were ranked by NES in descending order, and the pathways were visualized using aPEAR. Protein-protein interaction (PPI) data were retrieved using the STRING database (https://string-db.org/). The obtained interaction network was subsequently visualized using Cytoscape software (version v3.10.1).

### Prediction of potential transcription factors and interacting proteins

To identify transcription factors (TFs) regulating Protein A, we utilized the Harmonizome 3.0 platform (https://maayanlab.cloud/Harmonizome/), which integrates diverse datasets for gene and protein interaction prediction. Tools including ChEA, GEO Signatures, JASPAR, KnockTF, and MotifMap were applied to ensure comprehensive analysis. These tools leverage ChIP data, TF perturbation expression profiles, DNA-binding motifs, and enriched sequence motifs in regulatory regions. Predictions were integrated and refined by evaluating differentially expressed TFs (DE-TFs) from experimental datasets (|logFC|> 0, *p*-value< 0.05). DE-TFs were further categorized based on their relevance to LTBI or ATB, enabling the identification of condition-specific regulators of PD-L1 expression. Potential PD-L1 interactors were predicted using HitPredict (https://www.hitpredict.org/help.html), GeneMANIA (https://genemania.org/), and STRING (https://cn.string-db.org/). These tools combine data from high-confidence experiments, functional genomics datasets, and text mining to identify physical and functional PPIs. The predicted interactors were further assessed for differential expression across low/high-risk LTBI and ATB/low-risk LTBI groups (*p*-value< 0.05, |logFC| > 0). Context-specific interactors were identified, highlighting proteins potentially mediating PD-L1’s distinct roles in different disease states. Pathway enrichment analyses were conducted using the “ClusterProfiler” R package. Significance was determined with a *p*-value threshold of< 0.05. The results were visualized using the “dotplot” packages.

### Identification and evaluation of candidate compounds

Candidate small-molecule compounds targeting the identified hub genes were screened using the Drug Signatures Database (DSigDB) via the Enrichr platform (https://maayanlab.cloud/Enrichr/), with an adjusted *p*-value threshold of< 0.01 considered statistically significant (Yoo et al., 2015). DSigDB provides curated associations between drugs and gene expression profiles, enabling hypothesis-driven compound prioritization. As no experimentally resolved crystal structures for TCOF1 and LMO2 were available in the Protein Data Bank (PDB), predicted protein structures were obtained from the AlphaFold Protein Structure Database (https://alphafold.ebi.ac.uk/) and saved in PDB format. The corresponding ligand structures were downloaded from PubChem in 3D SDF format. Molecular docking was performed using CB-Dock2, an automated protein-ligand docking platform that incorporates binding cavity detection and template-guided docking (Liu et al., 2022). Docking results were subsequently visualized to evaluate binding pose and affinity.

### Construction and identification of L-Mtb

Dormant, lipid-rich Mycobacterium tuberculosis (L-Mtb) was generated from logarithmic-phase M. tuberculosis H37Rv cultures. Briefly, exponentially growing bacilli were transferred into 7H9 medium (BD Difco, Franklin Lakes, NJ, USA) supplemented with 10% ADC enrichment and 0.05% Tween-80, and incubated under oxygen-limiting conditions to induce dormancy. To establish the hypoxic environment, methylene blue was added as an oxygen indicator at a final concentration of 1.5 μg/mL, and the cultures were tightly sealed with Parafilm and overlaid with wax. The cultures were maintained at 37°C with shaking at 160 rpm until complete decolorization of methylene blue, typically within 21–28 days, indicating oxygen depletion and the establishment of a dormant phenotype. Dormant bacilli were identified using dual Auramine O-Nile Red staining. Bacterial smears were fixed in methanol, stained with 0.1% Auramine O at 37°C for 15 min to visualize acid-fast bacilli (green fluorescence, excitation 450–490 nm), and subsequently counterstained with 0.5 μg/mL Nile Red for 10 min at room temperature in the dark. Nile Red fluorescence selectively marked lipid-rich, non-replicating Mycobacterium tuberculosis, which are characteristic of the dormant state (Daniel et al., 2011; Kapoor et al., 2013; Vilchèze and Kremer, 2017).

### Establishment of dormant bacterial infection model

Bone marrow-derived macrophages (BMDMs) were isolated from 6-8-week-old C57BL/6 mice (SPF grade) and cultured in RPMI-1640 medium containing 10% fetal bovine serum (FBS) and 30 ng/mL M-CSF for 7 days at 37°C in 5% CO_2_. BMDM purity was confirmed by flow cytometry (**Supplementary Figure 1A, B**). BMDMs were infected with L-Mtb at a multiplicity of infection (MOI) of 5:1 for 4 h. Non-internalized bacteria were removed by three washes with pre-warmed RPMI-1640. The final wash supernatant was examined by acid-fast staining to confirm the absence of extracellular bacilli. Infection efficiency was verified by acid-fast staining of adherent cells. After 24 h of incubation, cells were lysed with 0.5% Triton X-100 on ice for 10 min. Released bacilli were collected by centrifugation (12, 000 × g, 5 min), smeared onto glass slides, and subjected to dual Auramine O-Nile Red staining. Auramine O labels metabolically active acid-fast bacilli (green fluorescence), whereas Nile Red selectively stains lipid-rich, non-replicating bacilli characteristic of the dormant state (red fluorescence). Fluorescence images were captured using a fluorescence microscope, and 100 bacilli were randomly counted per field in three independent experiments. The proportion of dormant bacilli was calculated as the dormancy ratio (Arbués et al., 2025), defined as:

The proportion of dormant bacteria (%)=Nile Red^+^ bacilli/Auramine O^+^ bacilli × 100%

### Reverse transcription-polymerase chain reaction

Total RNA was extracted from L-Mtb-infected bone marrow-derived macrophages (BMDMs) using TRIzol reagent (Ambion, Austin, TX, USA), and reverse transcription was performed with the Eastep^®^ RT Master Mix Kit (Promega, Madison, WI, USA), including a no-RT control. PCR amplification was conducted using 2× Taq PCR MasterMix (Solarbio, Beijing, China). The primers used were as follows: m-PD-L1: forward 5′-CAGCAACTTCAGGGGGAGAG-3′, reverse 5′-CATGCTCAGAAGTGGCTGGA-3′; m-GAPDH (internal control): forward 5′-TCACCATCTTCCAGGAGCGAGAC-3′, reverse 5′-TGAGCCCTTCCACAATGCCAAAG-3′.

PCR products were separated on 2% agarose gels and visualized under UV illumination. Band intensities were quantified using ImageJ software, and m-PD-L1 expression was normalized to GAPDH. To ensure the reliability of semi-quantitative analysis, RNA extraction was performed independently from the samples used for staining, and equal amounts of RNA were used for each reaction. All PCR products were electrophoresed on the same gel under identical exposure and staining conditions within the linear detection range. Each experiment was conducted in three independent biological replicates.

### Statistical analysis

Data were analyzed using SPSS (IBM) and GraphPad Prism 9.5.0. Normality was assessed using the Shapiro-Wilk test, and homogeneity of variance was evaluated with the Brown–Forsythe test. Two-group comparisons were performed using unpaired t-tests. For comparisons involving more than two groups, one-way or two-way ANOVA was conducted as appropriate, followed by Dunnett’s *post hoc* test to compare each treatment group with the control group. Results are presented as mean ± standard deviation (SD), and *P* < 0.05 was considered statistically significant.

## Results

### Inhibition of PD-L1 is associated with increased risk of TB

**Figure 2:**
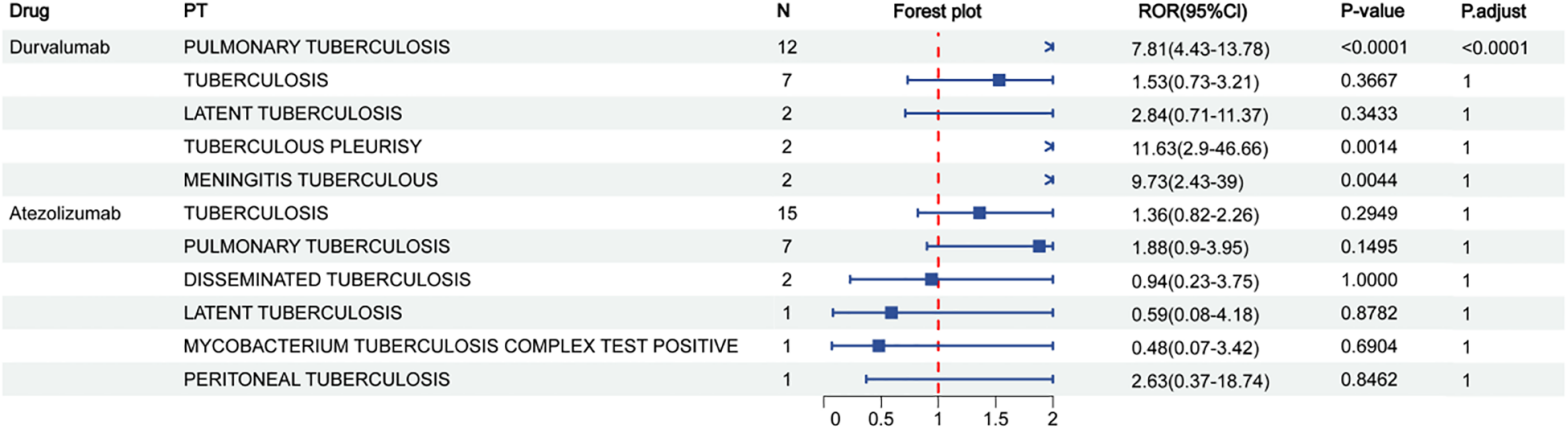
Reporting odds ratios (ROR) for durvalumab and atezolizumab in relation to different types of tuberculosis. N represents the sample size.

To investigate the clinical relevance of PD-L1 blockade in TB progression, we analyzed pharmacovigilance data from the FAERS database, focusing on tuberculosis-related adverse events (TB-AEs) reported from Q1–2004 to Q2 2024. Cases associated with concurrent immunosuppressive therapy were excluded from the analysis. However, patients with lung cancer or urothelial carcinoma, who inherently carry a higher TB risk due to their disease and cancer-related treatments, could not be excluded. Among the three FDA-approved PD-L1 inhibitors—durvalumab, atezolizumab, and avelumab—only durvalumab showed a statistically significant signal for TB-AEs (ROR = 7.81, 95% CI: 4.43–13.78), supported by other disproportionality metrics (PRR = 7.80, EBGM_05_ = 4.67, IC025 = 2.15) (**Figure 2**, **Supplementary Table 3**). Atezolizumab did not exhibit a significant signal, and no TB-AEs were reported for avelumab (**Supplementary Table 4**). These discrepancies may reflect differences in antigen epitope recognition or drug pharmacodynamics. Collectively, these findings align with the established notion that assessing latent tuberculosis infection before initiating immune-modulating therapies is essential for minimizing reactivation risk (Aguilar-Company et al., 2022), providing additional evidence from real-world pharmacovigilance data in the context of PD-L1–targeted treatment.

### PD-L1^high^ CD14^-^ CD16^+^ monocytes confer protection against early TB progression

**Figure 3:**
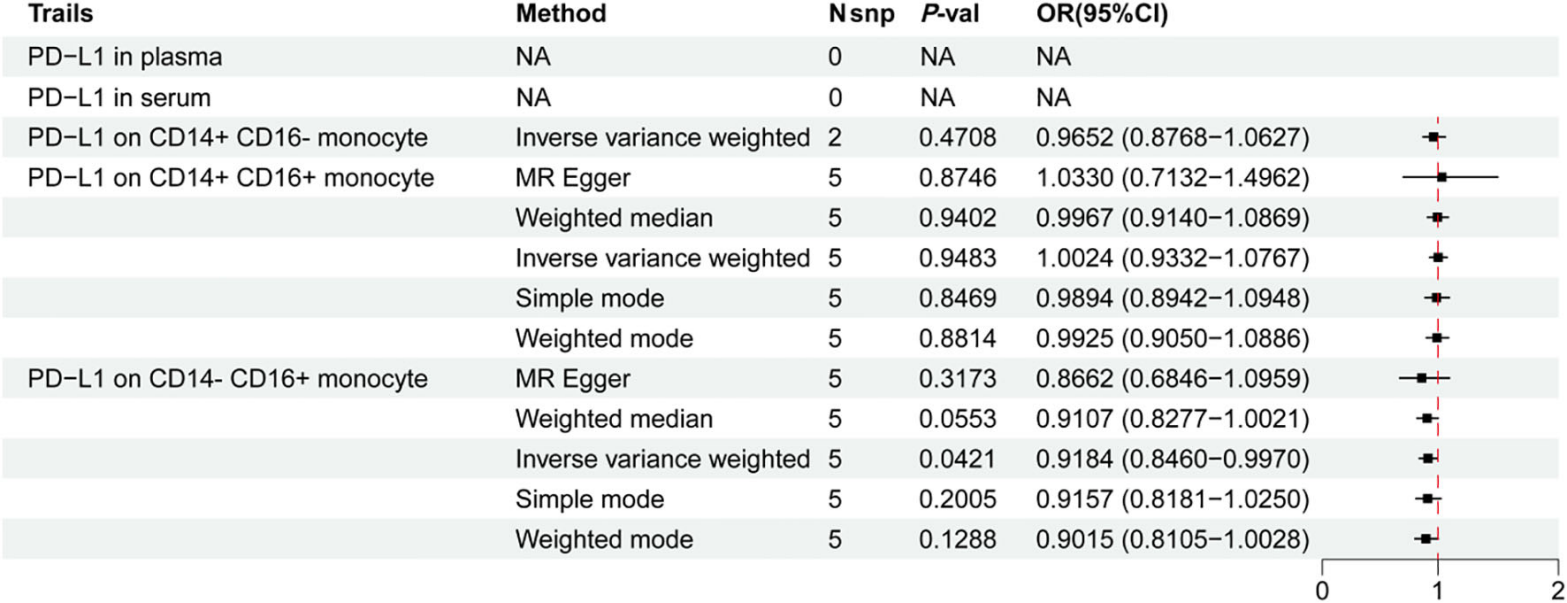
Causal effect of monocyte subset characteristics on the risk of early TB progression. Inverse variance weighting was the primary method used to determine these associations.

Although pharmacovigilance analyses revealed a strong disproportionality signal between PD-L1 inhibitor use and tuberculosis-related adverse events, such findings remain susceptible to potential confounders, including comorbidities and indication bias. To infer causality, we conducted MR using genetic instruments for PD-L1 expression (**Supplementary Table 5**), adjusted for key confounders including BMI, smoking, alcohol use, diabetes, and HIV status (*P* < 1×10^-5^). Notably, no significant associations were detected for PD-L1 levels in serum or plasma (**Figure 3**). However, genetically predicted high PD-L1 expression specifically in CD14^-^ CD16^+^ monocytes was significantly associated with a reduced risk of early TB progression (OR = 0.9184, 95% CI: 0.8460-0.9970, *P*_IVW_ = 0.0421), consistent across four additional MR models (**Figure 3**, and **Supplementary Figure 1A, B**). Sensitivity analyses revealed no heterogeneity or horizontal pleiotropy (*P* > 0.05), and leave-one-out analysis confirmed the stability of the results. Reverse MR ruled out reverse causation (**Supplementary Table 6, 7**, and **Supplementary Figure 1C, D**). MR-PRESSO further supported the protective role of PD-L1 on CD14^-^ CD16^+^ monocytes (*β* = -0.0544, *P* = 0.0164).

Together, these findings reveal a causal and cell-specific immunoregulatory role for PD-L1 in maintaining LTBI, emphasizing the risk of disrupting this axis during PD-L1-targeted therapies.

### PD-L1^low^ CD14^-^ CD16^+^ monocytes in high-risk individuals exhibit distinct immune-metabolic dysregulation

**Figure 4:**
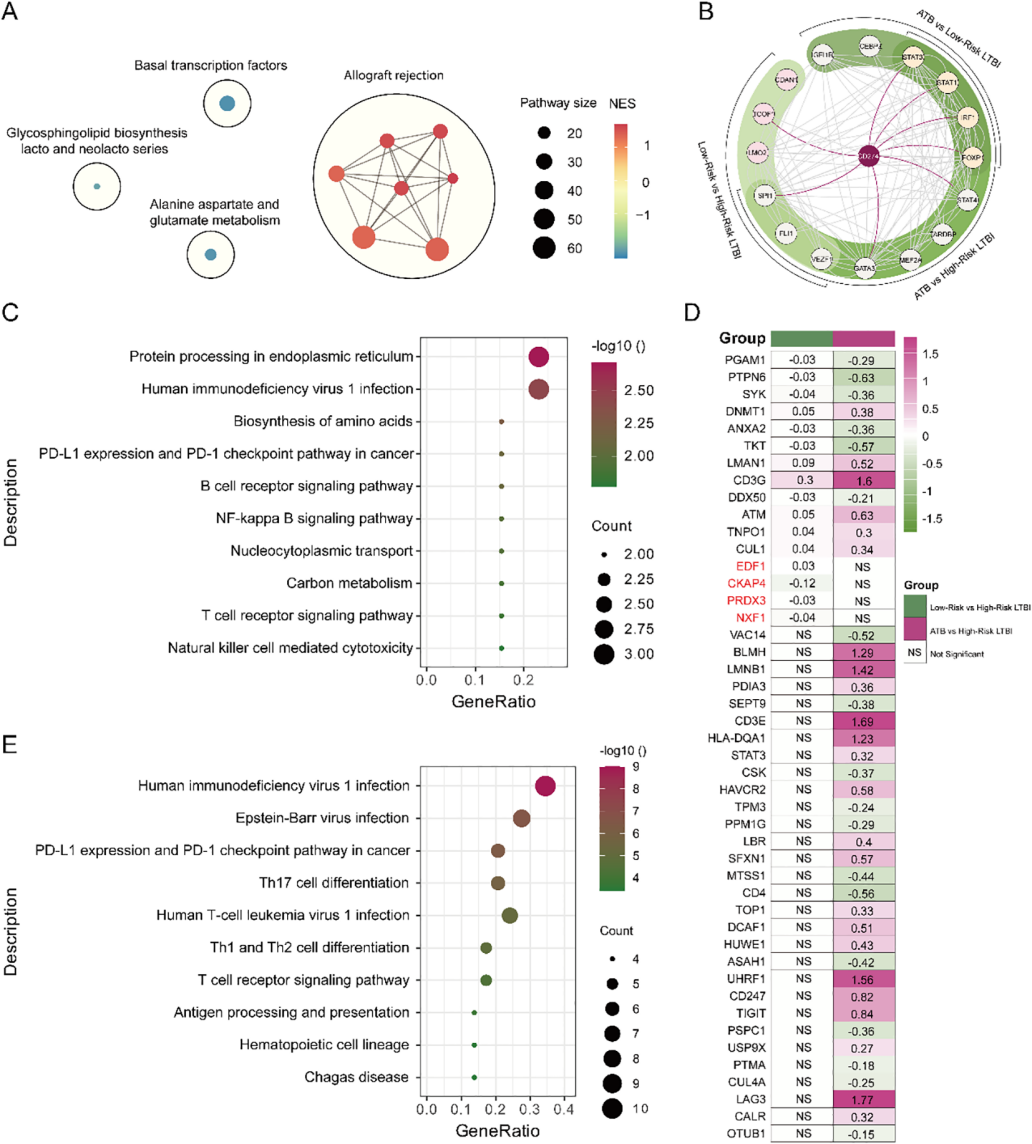
**(A)** Pathway interaction network of GSEA-KEGG results. Black circles indicate clusters of similar pathways involved in related biological processes. Line thickness reflects the degree of similarity between pathways, and labeled pathways represent the most representative ones within each cluster. **(B)** The Venn diagram highlights shared and unique transcription factors regulating PD-L1 between low-risk LTBI and ATB groups, while the PPI network displays interactions among these proteins. **(C)** KEGG enrichment analysis of PD-L1-interacting proteins reveals functional differences between low-risk LTBI. **(D)** KEGG enrichment analysis of PD-L1-interacting proteins reveals functional differences between ATB groups. **(E)** Expression levels of PD-L1-interacting proteins are compared between the two groups, illustrating significant differences associated with disease states.

To characterize the molecular features underpinning early TB progression risk, we profiled the transcriptomes of CD14^-^ CD16^+^ monocytes stratified by PD-L1 expression. Individuals with low PD-L1 expression (PD-L1^low^), representing the high-risk group, displayed marked transcriptional reprogramming. Gene set enrichment analysis revealed upregulation of metabolic pathways, including glycosphingolipid biosynthesis and amino acid metabolism (alanine, aspartate, glutamate) (**Figure 4A**, **Supplementary Table 8**). Conversely, immune effector pathways, such as antigen processing and presentation, cell adhesion, and NK cell–mediated cytotoxicity, were significantly downregulated. These findings suggest that PD-L1^low^ CD14^-^ CD16^+^ monocytes may drive early TB progression via immune suppression coupled with metabolic rewiring.

### Distinct transcriptional regulators of PD-L1 govern monocyte-mediated TB progression risk

We next identified transcriptional regulators (TFs) driving PD-L1 expression in CD14^-^ CD16^+^ monocytes under different risk states. Three TFs, CDAN1, TCOF1, and LMO2, were uniquely enriched in the high-risk group and absent in comparisons between LTBI and active TB (ATB), implicating them as specific mediators of reactivation susceptibility (**Figure 4B**, **Supplementary Tables 9, 10**). In contrast, SPI1, FLI1, and VEZF1 were shared by both high-risk and ATB groups, exhibiting a biphasic regulation pattern: upregulated in LTBI reactivation, but downregulated in progression to ATB (*P* < 0.05). This dynamic behavior suggests a transitional regulatory role. Additional TFs—STAT1, STAT3, IRF1, and FOXP1—were exclusive to the ATB group (**Supplementary Table 11**), aligning with their known roles in inflammatory amplification.

Protein interaction analysis further revealed 16 PD-L1-interacting proteins uniquely enriched in the high-risk group, including EDF1, CKAP4, PRDX3, and NXF1, which were functionally involved in immune pathways such as the PD-1/PD-L1 checkpoint, NK cell cytotoxicity, and NF-κB signaling (**Figures 4C, D**; **Supplementary Tables 12-15**). These proteins are likely key modulators of PD-L1’s immunoregulatory role in LTBI maintenance. By contrast, PD-L1 interactors in the ATB group were primarily enriched in pathways related to Th1, Th2, and Th17 cell differentiation (**Figure 4E**), indicating a context-dependent function of PD-L1 across disease stages. Together, these results emphasize that distinct PD-L1 regulatory networks operate in latent versus active TB, supporting the need for separate therapeutic strategies: e.g., targeting STAT1/3, IRF1, and FOXP1 for ATB, and CDAN1, TCOF1, and LMO2 for LTBI reactivation prevention.

### Identification of candidate small-molecule agents for LTBI treatment

To translate these findings into therapeutic opportunities, we queried the DSigDB database for compounds targeting the three reactivation-specific TFs. Ten candidate drugs were identified, alprostadil, ruthenium, pomalidomide, ganciclovir, captopril, lycorine, zidovudine, geldanamycin, aziridine, and acridine orange, predicted to interact with TCOF1 or LMO2 (**Table 2**). Notably, several of these compounds (e.g., ruthenium complexes, pomalidomide, aziridine) have reported anti-mycobacterial effects, while others possess known antiviral or immunomodulatory activity (Zhang et al., 2017; da Silva et al., 2018; Zhang et al., 2025).

**Table 2:** Prioritized small-molecule compounds potentially repurposed for LTBI intervention, with known indications and antimicrobial evidence.

Compound name	Adjusted p-value	Target genes	Primary indication	Clinical status^a^	Evidence for antimicrobial/anti-infective activity^b^	Binding affinity (TCOF1, kcal/mol)	Binding affinity (LMO2, kcal/mol)
Alprostadil	0.045	TCOF1; LMO2	Erectile dysfunction	Approved	Potential antimicrobial activity	–5.2	–5.3
Ruthenium	0.045	TCOF1	Experimental anticancer agent (Ru-based)	Preclinical/Clinical trials	Certain complexes show antimycobacterial activity	–	–
Pomalidomide^c^	0.045	LMO2	Multiple myeloma	Approved	Active against Mycobacterium tuberculosis	–	–6.0
Ganciclovir	0.045	TCOF1	Cytomegalovirus (CMV) infection	Approved	Primarily for CMV; limited broader antimicrobial scope	–5.3	–
Captopril	0.045	TCOF1	Hypertension, heart failure	Approved	Protective effects in pulmonary infections	–4.4	–
Lycorine	0.045	TCOF1; LMO2	Antiviral and anticancer research	Preclinical	Reported antifungal, antiviral, and antimalarial effects	–6.4	–6.2
Zidovudine	0.045	TCOF1	HIV/AIDS	Approved	Reduced pulmonary infection risk in HIV patients	–6.2	–
Geldanamycin	0.045	TCOF1	HSP90-targeted anticancer agent	Preclinical/Early trials	Demonstrated antibacterial activity	–5.9	–
Aziridine	0.045	TCOF1	Alkylating scaffold for anticancer agents	Preclinical/Early trials	Exhibits antimycobacterial activity	–2.1	–
Acridine Orange	0.045	TCOF1	Fluorescent dye; anticancer applications	Preclinical	Primarily used in cancer diagnostics and phototherapy	–5.5	–

a Clinical use status was retrieved from the FDA Drug Approvals and Databases (https://www.accessdata.fda.gov/scripts/cder/daf/index.cfm).

b see **Supplementary Table 16** for full reference list.

c Molecular docking for Pomalidomide could not be performed due to atomic incompatibility.

**Figure 5:**
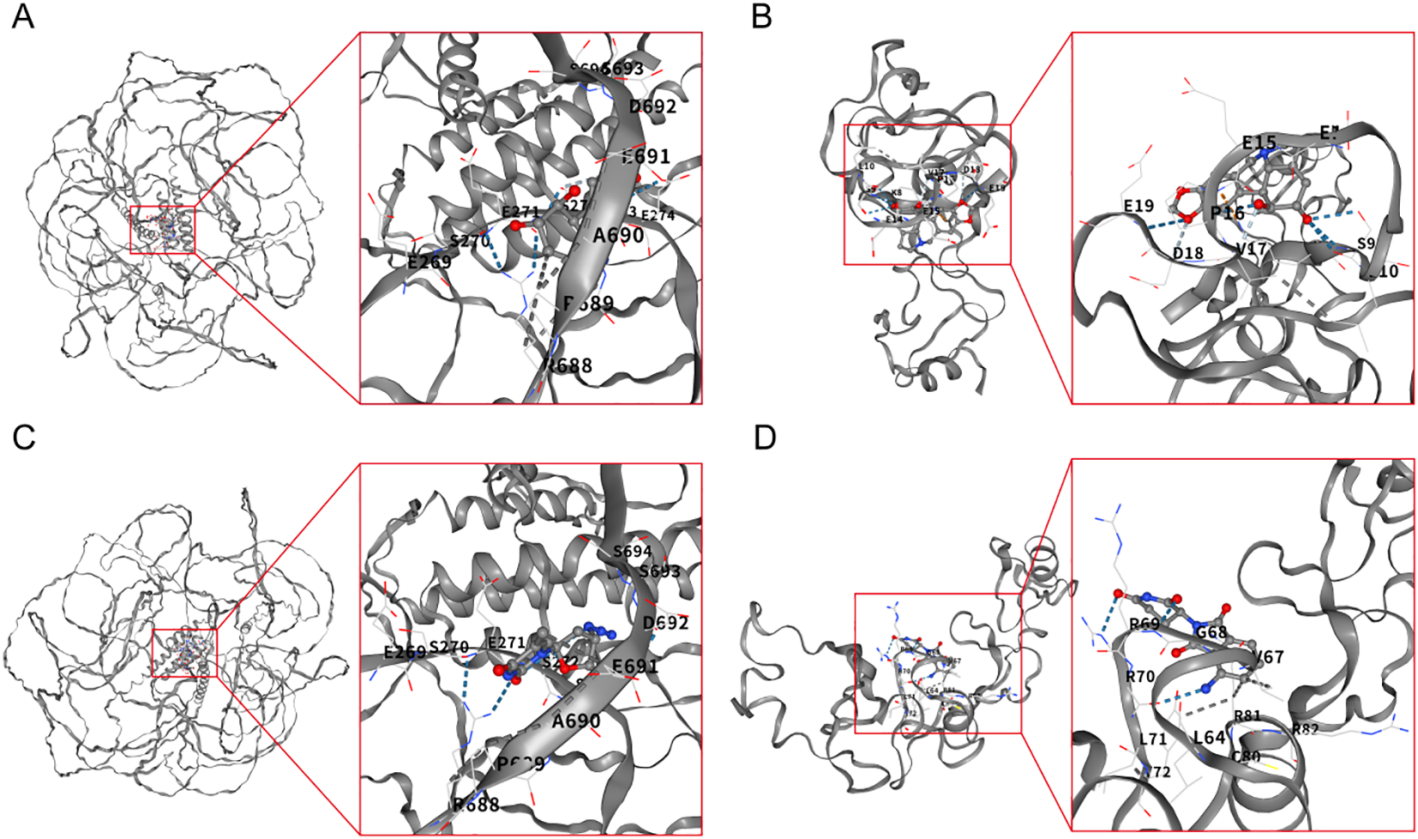
Molecular docking experiment. **(A)** Molecular docking model of Lycorine and TOCF1. **(B)** Molecular docking model of Lycorine and LMO2. **(C)** Molecular docking model of Zidovudine and TOCF1. **(D)** Molecular docking model of Pomalidomide and LMO2.

Molecular docking using CD-Dock2 revealed favorable binding energies (< –5.0 kcal/mol) for most compounds. Lycorine showed the strongest affinities to both TCOF1 (–6.4 kcal/mol) and LMO2 (–6.2 kcal/mol), followed by pomalidomide (–6.0 kcal/mol) and zidovudine (–6.2 kcal/mol), indicating strong target engagement (**Figures 5A–D**). These findings nominate several clinically relevant compounds as promising candidates for repurposing to prevent LTBI reactivation, warranting further preclinical validation.

### Lycorine suppresses LTBI reactivation and upregulates PD-L1 expression in macrophages

**Figure 6:**
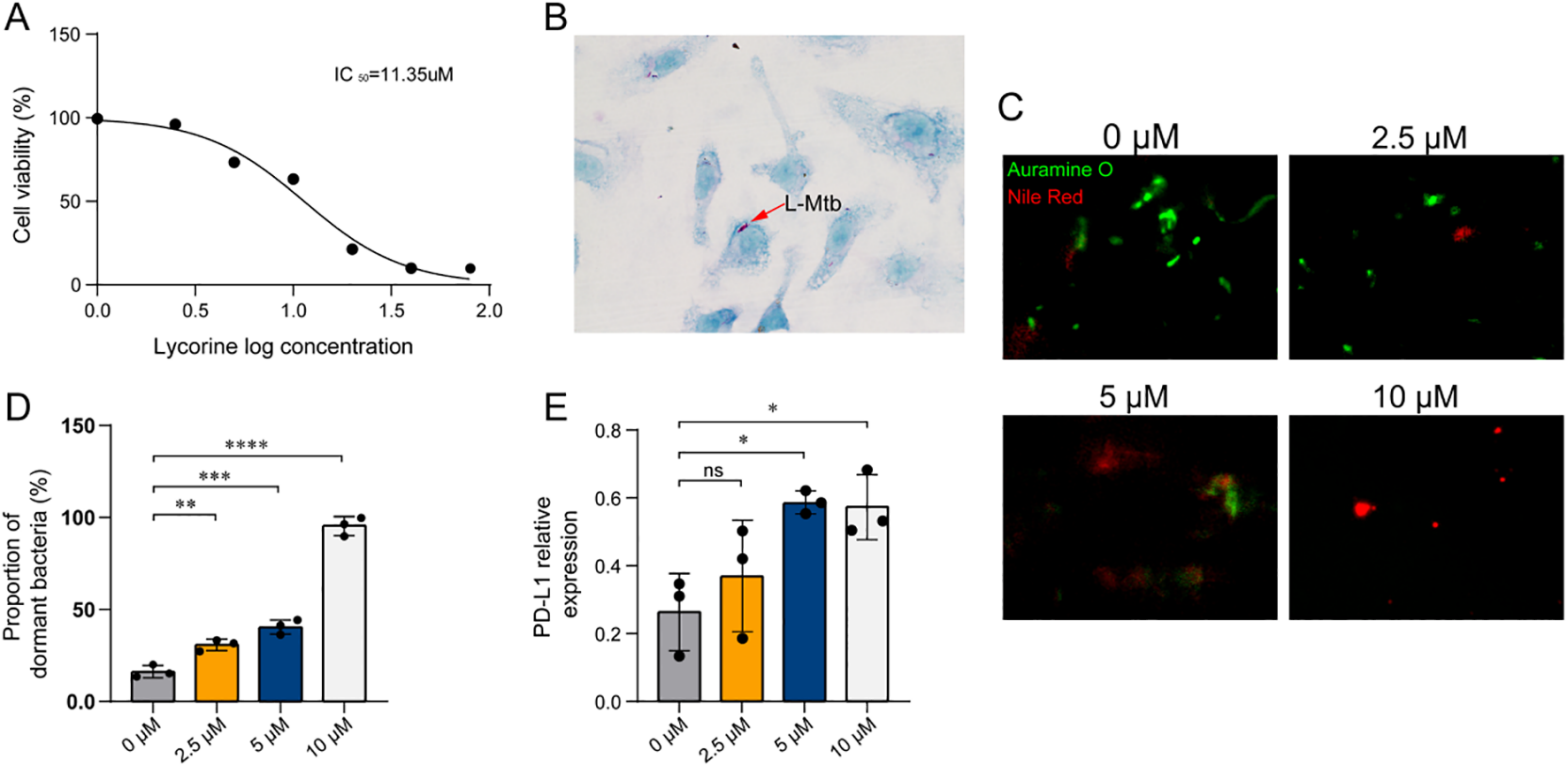
Lycorine effects on L-Mtb–infected BMDMs. **(A)** Cell viability assessed by CCK-8 assay following lycorine treatment. Three independent biological replicates were performed. **(B)** Identification of L-Mtb–infected BMDMs by acid-fast staining (1000× magnification). Arrows indicate acid-fast positive bacilli. **(C)** Auramine O–Nile Red dual staining of intracellular bacilli (1000×). Auramine O (green fluorescence) labels metabolically active, acid-fast bacilli, while Nile Red (red fluorescence) selectively stains lipid-rich dormant bacilli. The proportion of dormant bacilli was calculated as: % dormant = (Nile Red^+^ bacilli/Auramine O^+^ bacilli) × 100%. **(D)** Quantification of dual staining results. Dot plots show individual data points from three independent experiments. **(E)** Semi-quantitative RT-PCR analysis of PD-L1 mRNA expression in infected BMDMs. Relative expression was calculated as PD-L1 band intensity normalized to GAPDH. Dot plots show data from three independent experiments. Statistical analysis: One-way ANOVA was used for comparisons among multiple groups. *P < 0.05, **P< 0.01, ***P < 0.001, ****P< 0.0001. Colors of the bars/dots represent different lycorine concentrations for visual clarity only.

To explore the therapeutic potential of lycorine, a small-molecule compound with strong predicted binding affinity to TCOF1 and LMO2, we conducted a series of *in vitro* experiments targeting LTBI. We first evaluated the cytotoxicity of lycorine in BMDMs using CCK-8, with an IC_50_ of 11.35 µM. This indicated that lycorine is non-toxic at concentrations (0-10 µM) used in subsequent functional assays (**Figure 6A**).

A macrophage-based LTBI model was established by infecting BMDMs with dormant Mtb, confirmed by acid-fast staining (**Figure 6B**). Following infection, cells were treated with lycorine at concentrations of 0, 2.5, 5, and 10 µM. At 24 hours post-treatment, Auramine O-Nile Red dual staining was performed to quantify the proportion of dormant bacteria. Results showed that the proportion of dormant Mtb exhibited a dose-dependent increase with increasing lycorine concentrations, indicating that lycorine effectively suppresses the transition from latent to active infection (**Figures 6C, D**). Consistently, semi-quantitative RT-PCR analysis revealed that lycorine treatment led to a concentration-dependent increase in PD-L1 mRNA expression in infected BMDMs; notably, PD-L1 levels in the 10 µM lycorine group were significantly higher than those in both untreated infected controls and uninfected controls (**Figure 6E**). Although the mechanistic link between PD-L1 upregulation and suppression of LTBI reactivation remains to be elucidated, these findings suggest that lycorine exerts dual effects on infected macrophages—enhancing PD-L1-mediated immune regulation and limiting Mtb reactivation.

Taken together, lycorine demonstrates promising activity in suppressing LTBI reactivation *in vitro*, with potential to be developed as a lead compound for preventing TB reactivation in high-risk settings.

## Discussion

Tuberculosis latency reflects a dynamic equilibrium between host immunity and persistent infection, which is vulnerable to disruption by immune-modulating therapies. In this study, we identified PD-L1^high^ CD14^-^ CD16^+^ monocytes as a protective immune subset against LTBI reactivation. Integrating pharmacovigilance data, MR, transcriptomic profiling, and structure-based drug discovery, we demonstrate that PD-L1 suppression, particularly via the checkpoint inhibitor durvalumab, may destabilize host-monocyte interactions critical for maintaining TB latency. Notably, durvalumab is primarily used in the treatment of non-small cell lung cancer (NSCLC), whereas atezolizumab is used in NSCLC and other solid tumors, and avelumab is mainly indicated for urothelial carcinoma. Although lung cancer itself may constitute a potential risk factor for TB due to underlying lung pathology and frequent use of immunosuppressive therapies, current evidence does not clearly establish a direct causal link between lung cancer and TB onset; thus, the potential contribution of the underlying malignancy should be acknowledged without over-interpretation. These findings provide mechanistic insight into emerging clinical observations of TB reactivation in patients undergoing PD-L1-targeted immunotherapy.

To ensure conceptual clarity, we further emphasize that the genetic association analyses were based on the “tuberculosis early progression” dataset. This GWAS cohort defines cases as individuals who progressed to active TB within one year following recent exposure and controls as those who remained TST-positive but disease-free. Although this population captures early post-infection immune activation rather than long-term latency maintenance, it remains the only available genetic resource enabling evaluation of host susceptibility to TB progression. Therefore, the inferred genetic differences primarily reflect inherited determinants influencing the capacity to control early infection, offering mechanistic insights into pathways predisposing to or protecting against reactivation.

Previous pharmacovigilance studies using aggregated FAERS data have reported associations between PD-L1 blockade and TB reactivation36 but lacked stratification by specific inhibitors or TB disease phenotypes. Our analysis reveals a distinct signal for durvalumab, exclusively in pulmonary TB, whereas atezolizumab and avelumab show no such association. These discrepancies may arise from differences in epitope binding affinities, pharmacokinetics, or tissue distribution among PD-L1 inhibitors, though their precise mechanistic divergence requires further investigation (Lee et al., 2017).

Beyond drug-induced effects, the natural or spontaneous reactivation of latent TB—the form that accounts for the majority of LTBI progression—may involve similar or overlapping mechanisms. Importantly, PD-L1 expression on CD14^-^ CD16^+^ monocytes is known to increase during immune activation, including in active TB, and should not be interpreted directly as protective in that context. Our MR analysis, however, does not capture disease-induced PD-L1 upregulation but rather estimates the causal effect of genetically determined PD-L1 expression levels. Genetic instruments derived from healthy individuals indicate that a higher genetically predicted PD-L1 expression on CD14^-^ CD16^+^ monocytes is associated with a lower risk of progression to active TB. This observation suggests a protective genetic predisposition rather than an immunological effect occurring during active disease. We therefore speculate that suppression of PD-L1 on this monocyte subset, whether pharmacologically induced or occurring naturally, could compromise immune surveillance and granuloma integrity, facilitating Mtb reactivation. Although direct evidence linking PD-L1 modulation to spontaneous LTBI progression is currently limited, our results provide a framework for understanding how variations in monocyte-intrinsic PD-L1 expression may contribute to both drug-related and natural reactivation events.

Consistent with the MR inference, individuals in the LTBI cohort were stratified into “high” and “low” PD-L1” expression groups based on CD14^-^ CD16^+^ monocytes, revealing transcriptional and pathway differences coherent with the genetic findings. This stratification does not assume that low PD-L1 directly marks immune deficiency but instead reflects distinct intrinsic immune regulatory capacities potentially influencing LTBI reactivation risk. The subsequent pathway analyses, therefore, aim to explore mechanisms underlying this genetically supported protective tendency, rather than disease-state PD-L1 changes.

Mechanistically, PD-L1^low^ CD14^-^ CD16^+^ monocytes from individuals at high risk of LTBI reactivation displayed downregulation of immunosurveillance pathways, including antigen presentation and natural killer (NK) cell cytotoxicity, coupled with upregulation of metabolic programs, such as glycosphingolipid biosynthesis. This dual transcriptional rewiring suggests a shift toward immune quiescence and metabolic activation, creating a permissive niche for Mtb reactivation. As macrophage precursors, these monocytes likely influence granuloma integrity. Supporting this, conditional deletion of PD-L1 in macrophages exacerbates TB pathology in murine models (Qu et al., 2024). However, direct functional validation of monocyte-specific PD-L1 remains necessary to establish causality.

We further identified a distinct transcriptional signature that separates LTBI reactivation risk from ATB. Notably, CDAN1, TCOF1, and LMO2 were selectively enriched in high-risk latent states, whereas STAT1, STAT3, and IRF1 were specific to ATB, consistent with their established roles in TB immunity. Among them, STAT3 serves as a regulatory hub in CD14^-^ CD16^+^ monocytes during Mtb infection. In contrast, TCOF1 and LMO2 represent previously unrecognized regulators in the TB context.

Functionally, TCOF1 (Treacle) is involved in ribosome biogenesis, mitotic progression, and DNA damage response (Grzanka and Piekiełko-Witkowska, 2021), and also functions as a prognostic marker in triple-negative breast cancer (Hu et al., 2022). Notably, TCOF1 undergoes UFMylation upon rDNA double-strand breaks (DSBs), facilitating nucleolar cap formation and rDNA repair (Panichnantakul et al., 2024). Similarly, LMO2, a LIM-domain transcriptional regulator, promotes DSB repair via homologous recombination (Parvin et al., 2019). These roles are particularly relevant given that Mtb induces DSBs in macrophages by generating reactive oxygen species (ROS) and interfering with host repair mechanisms (Roca et al., 2022; Weindel et al., 2022; Zhang et al., 2024). Intriguingly, recent studies suggest that PD-L1 may participate in DSB repair (Shu et al., 2024). Together, these findings raise the hypothesis that TCOF1 and LMO2 may function upstream of PD-L1, coordinating genome stability and immune quiescence to preserve TB latency—although this remains speculative and warrants further mechanistic studies.

To explore the therapeutic potential of these regulators, we employed structure-based virtual screening and identified ten candidate compounds predicted to bind TCOF1 and LMO2. Several compounds—including lycorine, pomalidomide, and ruthenium complexes—have previously demonstrated anti-Mtb or immunomodulatory activity. For instance, pomalidomide restricts intracellular Mtb by modulating autophagy (Zhang et al., 2025), and ruthenium-based compounds show macrophage-penetrating anti-Mtb effects, sometimes comparable to first-line TB drugs (da Silva et al., 2017; da Silva et al., 2018). Additionally, madurastatin B3, featuring an aziridine moiety, exhibits potent antimycobacterial activity in BCG-infected THP-1 cells. A limitation of our approach is the lack of resolved protein structures for TCOF1 and LMO2 in the Protein Data Bank, necessitating reliance on AlphaFold-predicted models, which may reduce docking precision.

Among screened candidates, lycorine demonstrated the lowest predicted binding energy for both TCOF1 and LMO2, prompting functional validation. *In vitro*, lycorine dose-dependently upregulated PD-L1 expression in monocytes and inhibited reactivation of dormant Mtb, suggesting restoration of the PD-L1 axis as a therapeutic mechanism. Whether this effect is directly mediated through TCOF1/LMO2 remains to be determined. Nevertheless, lycorine’s dual functions—enhancing PD-L1 expression and directly suppressing Mtb reactivation—support its potential as a multi-targeted agent in LTBI management.

For translational development, future work should prioritize *in vivo* efficacy testing in murine LTBI models to validate lycorine’s protective role under physiological conditions, alongside assessments of systemic toxicity and pharmacokinetics. Combinatorial strategies with first-line anti-TB agents (e.g., isoniazid, rifampicin) are also worth exploring, given that lycorine’s immunometabolic mode of action may synergize with conventional bactericidal therapies.

In summary, our findings delineate a monocyte-intrinsic PD-L1 regulatory axis critical for TB latency, uncover TCOF1 and LMO2 as novel regulators within this axis, and propose candidate small molecules capable of modulating this network. By integrating genetic, transcriptional, and pharmacological evidence, we further clarify that the observed associations reflect a genetically determined predisposition influencing immune control rather than secondary effects of immune activation. These results are consistent with the broader understanding that evaluating latent tuberculosis infection prior to the initiation of immune-modulating therapy is essential for minimizing reactivation risk (Aguilar-Company et al., 2022), offering theoretical support from a systems-level perspective in the setting of PD-L1 blockade. Specifically, quantification of PD-L1 expression on CD14^-^ CD16^+^ monocytes, in combination with transcriptional profiling of TCOF1 and LMO2, may serve as a composite biomarker panel to stratify TB progression risk in patients undergoing PD-L1 inhibitor therapy. This approach could enable personalized prophylactic interventions, such as short-course anti-TB regimens or lycorine-based immunomodulation, thereby mitigating TB risk while preserving the anti-tumor efficacy of immune checkpoint blockade.

Interestingly, Underhill et al. recently reported that PD-L1 can function as an immune-activating receptor in addition to its conventional inhibitory role, promoting fungal recognition and antifungal immunity (Li et al., 2024). Consistently, our results show that high PD-L1 expression on CD14^-^ CD16^+^ monocytes correlates with reduced TB progression risk, suggesting that PD-L1 may exert protective effects during TB infection through regulation of innate immune activation, rather than solely through immune suppression. Notably, our pharmacological findings further support this hypothesis. Lycorine, identified through structure-based screening, not only upregulated PD-L1 expression in monocytes but also suppressed the reactivation of dormant M. tuberculosis, implying that restoration of the PD-L1 axis may represent a novel therapeutic mechanism for maintaining latency. This contrasts with PD-L1 inhibitors such as durvalumab, which destabilize this protective axis and predispose individuals to reactivation.

In contrast to conventional anti-TB agents (e.g., isoniazid, rifampicin) that primarily target bacterial metabolism, lycorine appears to act through host-directed immunomodulation, enhancing antimicrobial defenses without inducing overt inflammation. Beyond its anti-tubercular potential, lycorine has been reported to possess a wide spectrum of pharmacological activities—including anti-leukemic, anti-tumor, anti-angiogenic, antiviral, antibacterial, anti-inflammatory, and anti-malarial properties—with very low toxicity and mild side effects (Cao et al., 2013). Moreover, its capacity to reinforce PD-L1–dependent immune equilibrium distinguishes it from other small molecules such as pomalidomide or ruthenium complexes, which exhibit partial anti-mycobacterial activity but lack a defined mechanism for sustaining latency.

Together, these findings highlight lycorine as a promising host-directed adjunct that could restore immune homeostasis disrupted by PD-L1 blockade, offering a rational therapeutic avenue to prevent reactivation in high-risk individuals or patients undergoing immune checkpoint therapy.

## Data Availability

The original contributions presented in the study are included in the article/**Supplementary Material**. Further inquiries can be directed to the corresponding authors.
