# Supplementary material for: CD14- CD16+ monocyte PD-L1 prevents early tuberculosis progression and constrains reactivation under immune checkpoint therapy
Source: Front Cell Infect Microbiol. 2025 Dec 9;15:1684030. doi: 10.3389/fcimb.2025.1684030 (PMC12722520; doi:10.3389/fcimb.2025.1684030)
Supplement: Supplementary file 1 [file Table1.docx]

**
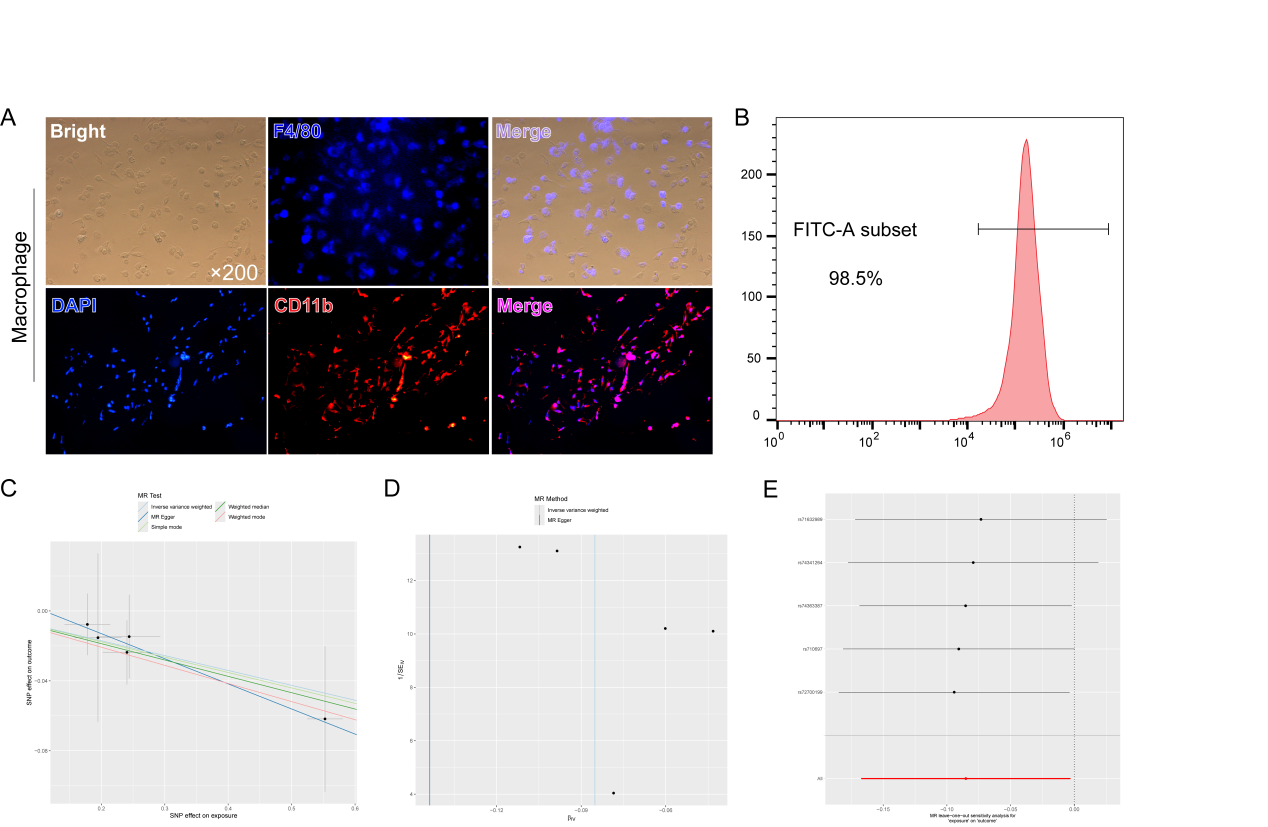
**

**Figure 1. Characterization of mouse bone marrow–derived macrophages and Mendelian randomization (MR) analysis of PD-L1 on CD14⁻CD16⁺ monocytes.** (A) Immunofluorescence staining of mouse bone marrow–derived macrophages (BMDMs) to assess purity (magnification ×200). (B) Flow cytometry analysis of BMDM purity, using FITC-conjugated anti-CD11b antibody. (C) Scatter plot of MR estimates depicting the causal effect of genetically determined PD-L1 expression on CD14⁻CD16⁺ monocytes on early progression of tuberculosis. (D) Funnel plot evaluating potential bias and heterogeneity in the MR analysis. (E) Leave-one-out sensitivity analysis assessing the influence of individual single-nucleotide polymorphisms (SNPs) on the causal estimate between PD-L1 expression on CD14⁻CD16⁺ monocytes and early progression of tuberculosis.
